# A droplet digital PCR detection method for rare L1 insertions in tumors

**DOI:** 10.1186/s13100-014-0030-4

**Published:** 2014-12-31

**Authors:** Travis B White, Adam M McCoy, Vincent A Streva, Joshua Fenrich, Prescott L Deininger

**Affiliations:** Tulane Cancer Center, 1430 Tulane Avenue, New Orleans, LA 70112 USA; Bio-Rad Laboratories, 750 Alfred Nobel Drive, Hercules, CA 94547 USA; Present Address: Eureka Genomics, 2000 Alfred Nobel Drive, Hercules, CA 94547 USA; Present Address: Division of Infectious Diseases, Boston Children’s Hospital and Harvard Medical School, 300 Longwood Avenue, Boston, MA 02115 USA

**Keywords:** L1, retrotransposon, droplet digital PCR, tumor

## Abstract

**Background:**

The active human mobile element, long interspersed element 1 (L1) currently populates human genomes in excess of 500,000 copies per haploid genome. Through its mobility via a process called target primed reverse transcription (TPRT), L1 mobilization has resulted in over 100 *de novo* cases of human disease and has recently been associated with various cancer types. Large advances in high-throughput sequencing (HTS) technology have allowed for an increased understanding of the role of L1 in human cancer; however, researchers are still limited by the ability to validate potentially rare L1 insertion events detected by HTS that may occur in only a small fraction of tumor cells. Additionally, HTS detection of rare events varies greatly as a function of read depth, and new tools for *de novo* element discovery are needed to fill in gaps created by HTS.

**Results:**

We have employed droplet digital PCR (ddPCR) to detect rare L1 loci in mosaic human genomes. Our assay allows for the detection of L1 insertions as rare as one cell in every 10,000.

**Conclusions:**

ddPCR represents a robust method to be used alongside HTS techniques for detecting, validating and quantitating rare L1 insertion events in tumors and other tissues.

**Electronic supplementary material:**

The online version of this article (doi:10.1186/s13100-014-0030-4) contains supplementary material, which is available to authorized users.

## Background

The human retrotransposon, long interspersed element 1 (L1) exists in over half a million copies per genome and constitutes 17% of genomic content [1]. The majority of these copies are nonfunctional relics that litter the genome; however, on average, approximately 100 L1 elements remain active in any given individual [1,2]. These active L1 elements mobilize in both germline and somatic tissues [3-11]. *De novo* L1 retrotransposition has been responsible for numerous germline diseases, as well as being implicated in tumorigenesis [8,10,12]. Notably, *de novo* L1 insertions have been identified in numerous cancer types including lung, colon, prostate, ovarian, and hepatocellular carcinoma through the use of high-throughput sequencing (HTS) technology [3-11].

Because tumors are often heterogeneous in genomic content, discovery and validation of *de novo* L1 insertion events detected by HTS in tumors can be problematic [13]. Validation statistics for HTS hits of *de novo* L1 somatic insertions have been reported to be as low as 67% [11]. One explanation for this fairly low rate of validation is tumor heterogeneity. Somatic L1 insertion events that occur late in tumorigenesis may represent a small minority of cells, and even insertion events that occur early in tumorigenesis may not be present in all tissue derived from that tumor. Some studies have had significantly higher validations, [3,7,10] but as methods develop to detect insertions present in smaller proportions of tumor cells, we can expect validation to become progressively more difficult.

Droplet digital PCR (ddPCR) has recently emerged as a robust tool to provide precise measurements of nucleic acid target concentrations [14,15]. In ddPCR, input DNA is partitioned, along with PCR reagents, into approximately 20,000 droplets as a water-in-oil emulsion within a single thermocycled reaction well [16]. Detection of target DNA relies on fluorogenic probes in a 5’-nuclease assay (TaqMan™) [17,18]. Briefly, an oligonucleotide probe, which anneals specifically to a target DNA within the primer binding sites, is included in the PCR with the primers. The probe is modified at the 5’ end with a fluorescent moiety, which is quenched in the intact probe by a modification at the 3’ end with a quencher moiety. The probe anneals to the target DNA during the annealing/extension step of the PCR. During extension of the primer that anneals to the same DNA strand as the probe, the 5’ to 3’ nuclease activity of *Taq* polymerase cleaves the probe, which separates the 5’-fluorescent nucleotide of the probe from the 3’ quencher, generating a fluorescent signal.

Sequestration of template DNA occurs in ddPCR, such that some droplets contain no copies and others one or more copies of the template target DNA [14,16]. Identification of template target DNA-containing droplets is achieved through fluorescence analysis of the droplets according to the 5’-fluorogenic probes used in the ddPCR. Droplets containing one or more target templates generate increased fluorescence compared to droplets containing non-target DNA. Thus, the quantification comes from the ability to essentially detect a single DNA template sequestered into a droplet through PCR amplification of the templates followed by counting of fluorescent droplets. The concentration of the input target DNA is calculated according to a Poisson distribution of template DNA molecules partitioned into the fluorescence-positive droplets [16]. Recent reports use ddPCR to successfully identify very rare alleles (that is, <1%) in heterogeneous tumor samples, making ddPCR an ideal method to apply for detection of rare *de novo* L1 insertion events [16]. Additionally, the utility of ddPCR over traditional qPCR methods has recently been examined [19].

Due to the high copy number of L1 sequence in the human genome, detection of specific polymorphic loci in a heterogeneous sample by traditional qPCR approaches is particularly difficult due to the high background signal created from nonspecific amplification from templates that do not contain the polymorphic L1. Partitioning of template DNA in ddPCR not only affords a reduction of this nonspecific background due to template dilution, it also allows an accurate determination of the concentration of the polymorphic L1 of interest in the input DNA. In this report, we apply ddPCR technology to the detection of rare L1 elements, allowing detection levels as low as one in every 10,000 cells. Our ddPCR assays incorporate L1 primers and probes that are common to each 5’ or 3’ junction ddPCR and specifically detect the youngest, actively mobile, L1Hs subfamiliy. By using universal L1 5’- and 3’-end primers and probes, paired with locus-specific flanking primers, this L1 detection method will prove useful as a way to rapidly identify *de novo* L1 insertion events in a heterogeneous tumor sample and to quantitate their frequency within an individual tumor sample. Additionally, L1 ddPCR allows heterozygote and homozygote loci to be easily distinguished through parallel detection of a second genomic locus.

## Results

**Figure 1:**
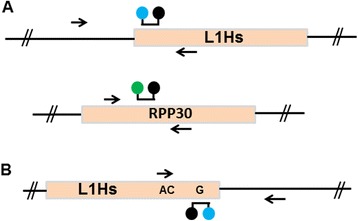
**Schematic of L1Hs droplet digital PCR (ddPCR) assays. (A)** A 5’-FAM labeled Taqman™ probe specific to the 5’ UTR of L1Hs is paired with an L1 5’ UTR-specific primer. The probe and primer anneal to complementary DNA strands. This primer/probe set can be used in conjunction with a unique genomic flanking primer to detect the 5’-insertion junction of specific full-length L1 elements in the human genome using ddPCR. A control assay using a primer and 5’-VIC labeled probe set specific to a housekeeping gene (RPP30) can be used in parallel to determine copy number. **(B)** A 5’-FAM labeled Taqman™ probe specific to the 3’ end of L1Hs is paired with an L1Hs-specific primer. The probe and primer anneal to complementary DNA strands. This primer/probe set can be used in conjunction with a unique genomic flanking primer to detect the 3’ insertion junction of specific L1Hs elements in the human genome using ddPCR.

For validation or discovery of *de novo* L1 insertion events, we designed assays to detect either the 5’- or 3’-insertion junctions at specific genomic loci. The core of each assay is a single primer and probe specific to the youngest L1 subfamily, L1Hs [2]. One primer and probe set is located at the 3’ end of L1Hs (Table 1; 3’ L1Hs primer, 3’ L1Hs probe), which may be used to detect both full-length and truncated L1Hs elements when paired with an appropriate locus-specific primer (Figure 1). The other primer and probe set is located at the 5’ end of L1Hs (Table 1; 5’ L1Hs primer, 5’ L1Hs probe) to detect full-length L1Hs 5’-insertion junctions when paired with an appropriate locus-specific primer (Figure 1). Amplification of the locus-specific L1Hs 5’- or 3’-insertion junction generates FAM fluorescence through nucleolytic cleavage of the annealed L1Hs-specific probe by *Taq* polymerase. For each experiment, a threshold of fluorescence is set relative to negative controls to measure the quantity of droplets that do or do not contain template target DNA. Through separation of DNA templates for PCR in up to 20,000 droplets and measurement of fluorescence for each droplet at the terminal plateau phase of PCR, ddPCR-based L1 detection is capable of a high degree of discrimination that is not possible with standard TaqMan™ assays [14]. Additionally, L1 ddPCR assays can be multiplexed with control ddPCR assays for housekeeping genes such as RPP30 to allow for accurate copy number determination [20].

**Table 1:** **Primers and probes used in this study**

**Primer/Probe Name**	**Sequence**
5’ L1Hs Primer	5’ GGAAATGCAGAAATCACCGTCTTC 3’
5’ L1Hs Probe (Chr 15)	5’ FAM AGGAACAGCTCCGGTCTACAGCTC BHQ1 3’
5’ L1Hs Probe (other loci tested)	5’ FAM AGGAACAGC/ZEN/TCCGGTCTACAGCTC IABkFQ 3’
5’ Chr15 AC216176 Locus Primer	5’ GTGGACAAAGAAAAGCATCCTTGAT 3’
RPP30 Forward Primer	5’ GATTTGGACCTGCGAGCG 3’
RPP30 Reverse Primer	5’ GCGGCTGTCTCCACAAGT 3’
RPP30 Probe	5’ VIC CTGACCTGAAGGCTCT MGB 3’
5’ Chr4 esv3475 Locus Primer	5’ CCACATGGTATAAGATAAAAACACGAG 3’
5’ Chr4 esv4912 Locus Primer	5’ CTAAGCAATGGAGGAAAATATCG 3’
3’ L1Hs Primer	5’ GGGAGATATACCTAATGCTAGATGACAC 3’
3’ L1Hs Probe	5’ FAM ATTATACTCTAAGTTTTAGGGTACATGTGCACATTGTGC BHQ1 3’
3’ Chr15 AC216176 Locus Primer	5’ TCTATAAGCAGTGGAAGCACATG 3’
3’ Chr4 esv3475 Locus Primer	5’ GACCAATTTTTTTTTGCCTGTACTGAC 3’
3’ Chr4 esv4912 Locus Primer	5’ TGCATTTTGAGTTAATTTTTGTACATGGTG 3’

Because they exist in high genomic copy number, L1 elements can contribute to significant background signal in PCR-based assays. In our assay designs, the L1Hs 5’ and 3’ end probes anneal to the same DNA strand as the locus-specific primer in each assay to ensure fluorescent signal is generated by extension of the primer at the L1-occupied chromosomal locus (Figure 1). This minimizes fluorescent signal arising from linear extensions off primers annealed at the numerous other genomic L1 loci. There is, however, still the possibility of amplification of two inverted L1Hs elements by two L1-specific primers resulting in background fluorescence in negative samples.

**Figure 2:**
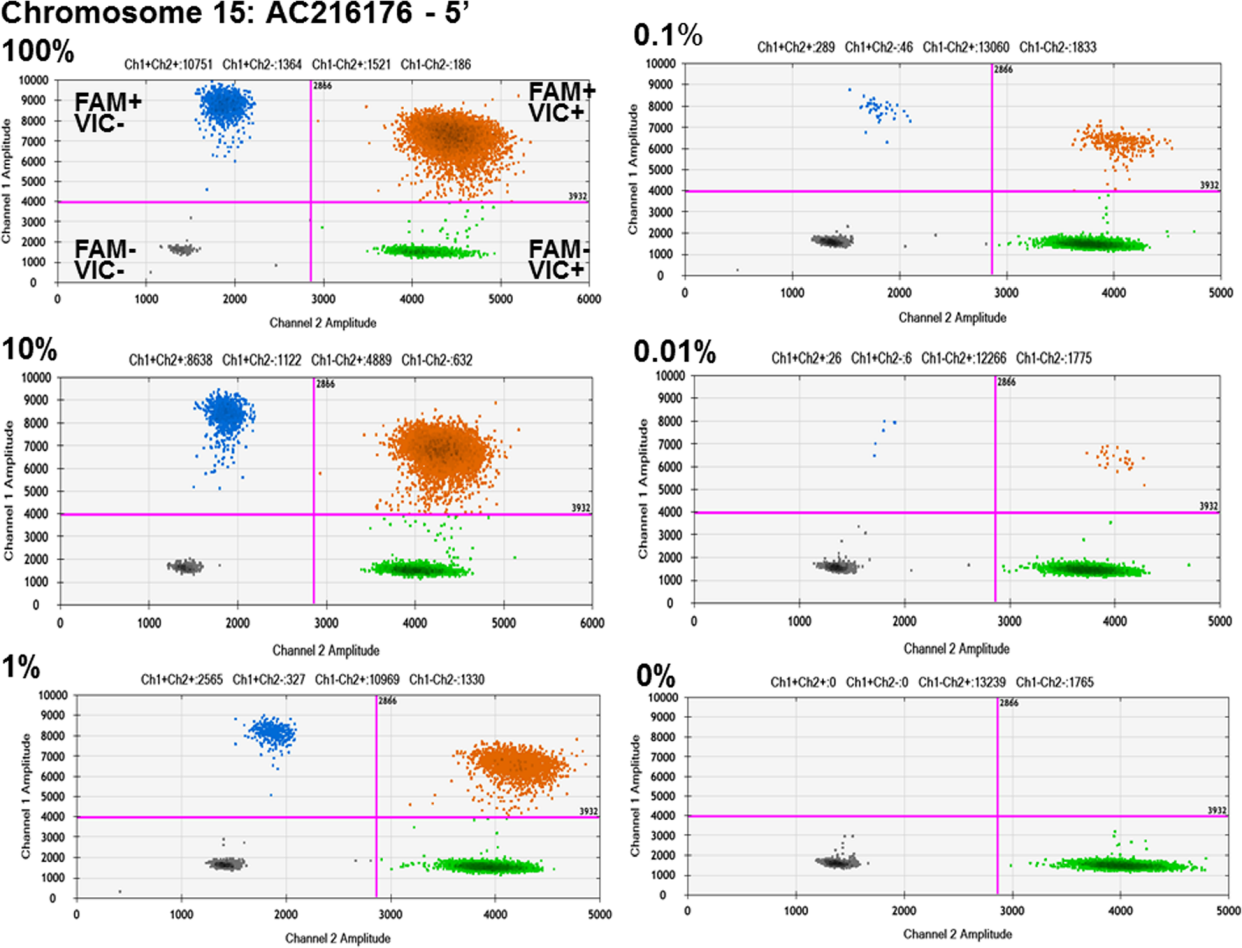
**Detection of chromosome 15 AC216176 L1Hs by the 5’ junction droplet digital PCR (ddPCR) assay.** Each panel represents a single ddPCR experiment whereby a DNA sample (defined below) is segregated into individual droplets and assessed for the presence of the L1 locus (FAM) and RPP30 locus (VIC) using two different fluorophores in Taqman™ assays (see Figure 1). The FAM and VIC fluorescence for each droplet is plotted as a data point on each graph. FAM fluorescent signal (Channel 1) is plotted on the y-axis and VIC fluorescent signal (Channel 2) is plotted on the x-axis. The droplet threshold for each fluorophore used is indicated by the magenta lines, determining whether a droplet is considered positive or negative for either FAM or VIC fluorescence. The positive or negative fluorescence assessment for each quadrant is labeled accordingly for the plot describing the experiment with 100% GM01632 DNA. The blue dots represent individual droplets that contain at least one copy of the L1 locus tested but not the RPP30 locus (FAM positive, VIC negative), the green dots represent droplets that contain at least one copy of the RPP30 gene and not the L1 locus (VIC positive, FAM negative), and the orange dots represent droplets that contain at least one copy of both the RPP30 gene DNA and the L1 locus tested (positive for both FAM and VIC). We tested 160 ng of *BsaJI*-digested genomic DNA from GM01632 cells, which are homozygous for the polymorphic L1 element (100%), and tenfold dilutions of this same sample as a mixture with *BsaJI*-digested genomic DNA from GM01631 cells, which do not have this polymorphic L1 insertion (10%-0.01%), thus keeping the total input genomic DNA constant for each ddPCR. Additionally, as a negative control, 160 ng of *BsaJI*-digested genomic DNA from GM01631 cells was tested (0%).

**Figure 3:**
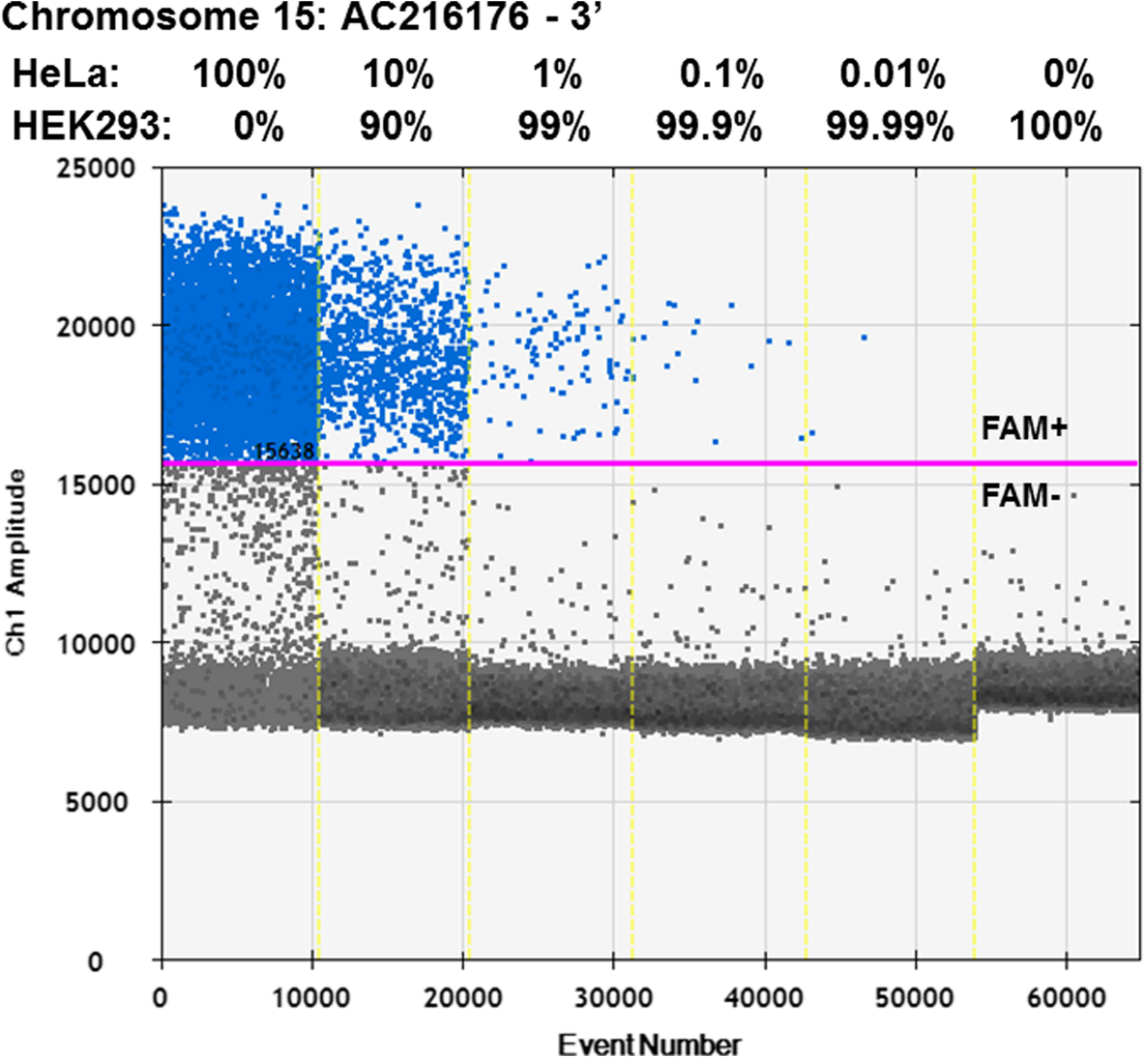
**Detection of chromosome 15 AC216176 L1Hs by the 3’ junction ddPCR assay.** The L1Hs 3’ junction ddPCR assay uses a L1-specific primer, L1-specific 5’-FAM labeled Taqman™ probe, and a locus-specific primer near the Chromosome 15 AC216176 3’-insertion junction, as shown in Figure 1B. The FAM fluorescent signal (Ch 1) for each droplet is plotted on the y-axis for each of the ddPCR experiments, which are separated by a dotted yellow line, with input DNA indicated above each experiment. Each droplet is cumulatively counted as an ‘Event Number’ for the ddPCR experiments analyzed in tandem, and plotted along the x-axis. The positive droplet fluorescence threshold is indicated by the magenta line, which determines whether a droplet is considered positive or negative for FAM fluorescence. Thus, the blue dots represent individual droplets that contain at least one copy of the L1 locus tested. We tested 200 ng of *BamHI*-digested genomic DNA from HeLa cells, which contain the polymorphic L1 element, and tenfold dilutions of this same sample as a mixture with *BamHI*-digested genomic DNA from HEK293 cells, which do not have this polymorphic L1 insertion. Percentages given reflect the amount of input DNA with 100% corresponding to 200 ng of DNA. This assay robustly detects the 3’-insertion junction of the polymorphic full-length AC216176 L1Hs element when present in the genomic DNA from a cell line positive for that polymorphism (HeLa 100%), but not in a cell line negative for that polymorphism (HEK293 100%). L1-positive droplets are observed at dilutions as low as 0.01% of the DNA with this assay.

We were able to generate L1-specific primers and probes that target young L1 insertions and result in only a minimal degree of non-specific background (Table 1, Figures 2 and 3). We developed a ddPCR assay for the L1 5’ end to detect *de novo* full-length L1 insertion events. Using a known polymorphic full-length L1Hs on Chromosome 15 (AC216176; [21]) as a model for our assay, we were able to successfully design a ddPCR assay that is able to robustly detect a specific L1Hs 5’-insertion junction known to be homozygous for the polymorphic L1 element in the cell line tested (Figure 2). To determine the limit of sensitivity of our ddPCR assay, we performed tenfold dilutions of this sample as a mixture with DNA from a sample known to be negative for the insertion, thus keeping the total input genomic DNA constant for each ddPCR. Detection of RPP30 by VIC fluorescence is consistent in each dilution experiment. This analysis allowed us to determine that the limit of sensitivity of our assay is as low as one positive cell in 10,000 total cells (0.01%) (Figure 2).

To assay specific 3’-insertion junctions of polymorphic L1 elements by ddPCR, we designed primers and probes unique to the 3’ end of the youngest L1Hs subfamily, which constitute the vast majority of L1 elements capable of retrotransposition [2]. The 3’ end of the 3’ L1Hs primer makes use of an AC dinucleotide at position 5926 of L1Hs, which gives the primer specificity to only these youngest L1 elements. Thus, although the primer can probably anneal to a significant portion of genomic L1 elements, it will only be able to prime DNA synthesis from these actively mobile, and therefore most interesting, L1 elements. Additionally, the L1 3’-end probe makes use of a G nucleotide at position 6011 of L1Hs, making it also specific for only the youngest L1 elements [2,22,23].

**Figure 4:**
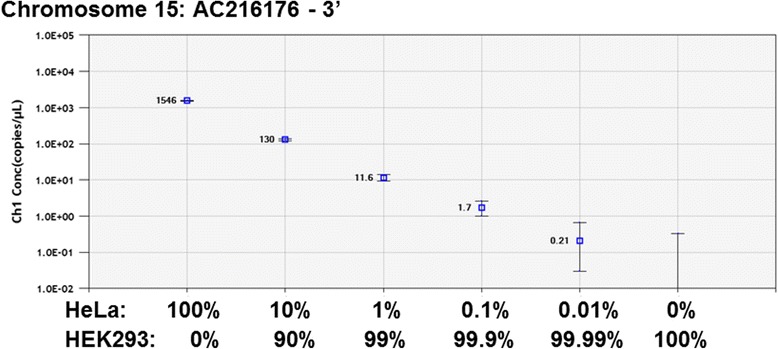
**Concentration plot of chromosome 15 AC216176 L1Hs by the 3’ junction droplet digital PCR (ddPCR) assay.** The input DNA concentrations in copies/μl (Ch1 Conc) for the ddPCR experiments described in Figure 3 were calculated by the QuantaSoft Analysis Software.

For the 3’ junction ddPCR experiment, we investigated the same known polymorphic full-length L1Hs on Chromosome 15 used as a model for our 5’-junction assay (AC216176; [21]). Using this primer and probe set, we were able to robustly detect a single, specific L1 3’-insertion junction with only minimal background (Figure 3). We additionally performed a dilution experiment as described above and were able to detect the L1 3’-insertion junction to one positive cell in every 1,000 total cells (0.1%) (Figures 3 and 4). Because establishment of the polymorphic L1 detection limit was our goal in these experiments, this ddPCR did not include RPP30 detection. Additionally, we showed that the 3’ end L1 primer and probe sets are specific to young L1 elements, as they do not amplify a known older (L1PA4) genomic L1 (data not shown).

To show that the L1-specific primers and probes may be used to detect the 5’- and 3’-insertion junctions of multiple polymorphic loci, locus specific primers were designed to detect the 5’ and 3’ junctions of two other known polymorphic full-length L1Hs elements (Additional file 1: Figure S1, Additional file 2: Figure S2, Additional file 3: Figure S3, Additional file 4: Figure S4, Additional file 5: Figure S5, Additional file 6: Figure S6, Additional file 7: Figure S7, Additional file 8: Figure S8). Detection of both 5’- and 3’-insertion junctions for a polymorphic element on Chromosome 4 (Database of Genomic Variants ID: esv3475, [24,25]) was sensitive to one positive cell in 1,000 total cells (0.1%) (Additional file 1: Figure S1, Additional file 2: Figure S2, Additional file 3: Figure S3, Additional file 4: Figure S4). Likewise, detection of both 5’- and 3’-insertion junctions of another polymorphic element on Chromosome 4 (Database of Genomic Variants ID: esv4912, [24,25]) was sensitive to one positive cell in 1,000 total cells (0.1%) (Additional file 5: Figure S5, Additional file 6: Figure S6, Additional file 7: Figure S7, Additional file 8: Figure S8).

## Discussion

Recent advances in detection of *de novo* L1 integration events by HTS have resulted in an increased understanding of the potential role L1 elements might play in the development of tumors. To date, L1 insertions have been detected by HTS in five different cancer types, and many of these insertions have been fully validated by traditional PCR-based strategies [6-11]. There are many more *de novo* L1 insertions, however, that have been detected through the use of HTS, but have not been able to be successfully validated. One likely explanation for this discrepancy is the genomic heterogeneity associated with tumors.

HTS technology has afforded researchers the ability to identify extremely low-frequency events that are difficult to validate by traditional PCR-based methods due to a high rate of background signal. *De novo* L1 insertions in tumors can often be classified as low-frequency events for a number of reasons. First, it is often difficult to fully separate normal adjacent tissue from tumors, with tissue dissected from certain tumor types sometimes containing a greater fraction normal than cancerous tissue [26]. Second, the timing of L1 mobilization in tumors has not been fully established. If L1 insertions occur at late stages in the development of a tumor, they will only be represented in a small fraction of the cells that compose the tumor. In this case, it remains very likely that such *de novo* L1 insertion events would be detected by some HTS studies, but would not necessarily be detectable by traditional PCR.

Droplet digital PCR (ddPCR) has proven itself capable of detecting extremely low frequency events [27]. In this study, we report the ability of a ddPCR assay to detect an L1 insertion event in as few as 0.01% to 0.1% of cells. This assay has a minimal level of background signal, which is surprising given the prolific nature of the L1 template in the human genome. The most likely source of background signal is the high level of L1Hs 3’ ends (approximately 5000 matching the 3’ L1Hs primer, but 500,000 with a partial match), resulting in off-target amplification between two L1-specific primers. Regardless of this, we are able to robustly detect an L1-positive signal in a low fraction of cells. Our ddPCR assay is not only a robust, straightforward tool to validate L1 insertion events detected by HTS from tumors, but is also capable of quantifying the fraction of cells in the tumor, or other material, that have that particular insertion.

Tumor cells undergo constant evolution and produce subclonal populations of cells, each containing different signatures of genomic rearrangements [28]. These chromosomal aberrations may serve as biomarkers for minor subclonal populations that harbor the capacity for relapse [28]. Indeed, there is a major effort to use HTS data to describe the subclonal genomic constituency of tumors and identify biomarkers for invasive subclonal populations of cells [29,30]. In addition to the validation of unique L1 insertions identified by HTS of tumors, the assays described here may be used to track and quantify mosaic L1 loci used as biomarkers for subclonal populations of cancer cells and, if an L1 insertion is unique to an individual’s identified cancer, establish a minimum level of residual disease detection.

Detection of rare alleles in the human population such as single-nucleotide polymorphisms, small insertions or deletions, or mobile element polymorphisms allows determination of disease-causing candidate genomic loci through association studies and shows regions of our genome that have been subject to selective pressures [31,32]. Establishment of rare allele frequencies through individual genotyping is a laborious and expensive process that can be overcome through methods that interrogate pools of human genomic DNA [33,34]. Our assay may be used as a means of establishing rare allele frequencies, in the range of 0.01%, in pools of human genomic DNA. Detection of the rare *BRAF* V600E allele has been previously demonstrated by ddPCR [14].

## Conclusions

Retrotransposition of long interspersed element 1 (L1) in human germline and somatic cells contributes to genomic variation in human populations and is implicated in tumorigenesis. In this study, we designed droplet digital PCR (ddPCR) assays to detect rare L1 insertion events in heterogeneous human genomic DNA samples. Traditional qPCR methods are unable to confidently discern rare target DNA sequences among input DNA as complex as a human genome due to low-chance priming events that cause background signal and lead to false-positive determinations. This effect is exacerbated when the target DNA involves L1 sequence, which occupies approximately 17% of the human genome. Using universal 5’ and 3’ L1 primers and probes in ddPCR, paired with a locus-specific primer near the assayed insertion site, we detected polymorphic L1 5’ and 3’ junctions in genomic DNA from a heterogeneous sample when as few as 0.01% of the cells contained the polymorphic L1. The ability to confidently detect and simultaneously quantify the level of a L1 insertion locus in a mosaic sample, such as tumor biopsy genomic DNA, will allow rapid validation of high-throughput sequencing data on *de novo* L1 insertions for a given sample, establishment of a minimum of residual disease detection for a cancer cell-specific L1 insertion, or sampling of pools of human genomic DNA for rare L1 allele detection.

## Methods

### Selection of L1 loci and primer/probe design

Polymorphic L1 elements were detected from genomic DNA from fibroblast cell lines GM01630, GM01631 and GM01632 (Coriell Institute; Camden, NJ, USA) using Sequencing Identification and Mapping of Primed L1 Elements (SIMPLE) (VAS, unpublished data). These polymorphic elements were previously confirmed by PCR. An identified polymorphic L1Hs locus was selected on the basis of identity to the L1Hs consensus sequence: a Chromosome 15 full-length L1Hs locus (AC216176; [21]). Additional polymorphic L1 loci tested were chosen among previously characterized polymorphic full-length L1 elements on Chromosome 4 (Database of Genomic Variants ID: esv4912, esv3475 [24,25]), and were assayed on the basis of identity to the L1Hs consensus sequence. Probes and primers were designed to match either the 5’ or 3’ end of the L1Hs consensus sequence (Table 1). The L1Hs 3’ primer and probe incorporate diagnostic nucleotides specific to L1Hs that are not present in older L1 elements. These L1Hs-specific primer/probe sets can be paired with a unique flanking primer specific to the genomic region of interest for detection of the 5’- and 3’-insertions junctions. Primers and probes were synthesized by Integrated DNA Technologies (Coralville, IA, USA), with the exception of those used for detection of RPP30 (Applied Biosystems now Life Technologies; Grand Island, NY, USA) (Table 1).

### Droplet digital PCR reaction conditions

Genomic DNA from fibroblast cell lines was extracted using the DNEasy Blood and Tissue Kit (Qiagen; Germantown, MD, USA). The 5’ junction ddPCR assays were performed in 20-μL reactions using ddPCR Supermix for Probes (Bio-Rad; Hercules, CA, USA) and 150 to 200 ng of *BsaJ*I- or *BamHI-*digested input DNA. Restriction enzyme digestions were done according to manufacturers’ protocol (New England BioLabs; Ipswitch, MA, USA). In the 5’ junction ddPCR assays, 900 nM 5’ L1Hs primer, 900 nM locus-specific primer, and 250 nM 5’ L1Hs probe was used. The 5’ junction ddPCR assays for the Chromosome 15 AC216176 locus included detection of the housekeeping gene RPP30 with 900 nM of each RPP30-specific primer and 250 nM RPP30 probe. Because the two loci are not linked, each droplet has a probability of being positive for either one of the loci, and some droplets will be either negative or positive for both. The relationship between presence/absence of each locus in a droplet is defined by the Poisson distribution and allows robust, digital quantification of the two loci relative to one another. Droplet generation was performed as per the manufacturer’s instructions. Cycling conditions were 95°C for 10 min, followed by 40 cycles of 94°C for 30 seconds and 64°C for two minutes, and then a final 10-min incubation at 98°C. Droplet reading was performed on a QX100 ddPCR droplet reader (Bio-Rad; Hercules, CA, USA) for the Chromosome 15 AC216176 locus, a QX200 ddPCR droplet reader (Bio-Rad; Hercules, CA, USA) for the other loci tested and analysis was done using QuantaSoft Analysis software (Bio-Rad; Hercules, CA, USA).

The 3’ junction ddPCR assays were performed in 20-μL reactions using ddPCR Supermix for Probes (No dUTP) (Bio-Rad; Hercules, CA, USA) and 200 ng *BamHI*-digested input DNA. In the 3’ junction ddPCR assay of the Chromosome 4 esv4912 polymorphic L1, 900nM of 3’ L1Hs primer, 900nM of locus-specific primer, and 200nM of 3’ L1Hs probe was used. In all other 3’ junction ddPCR assays, 900nM of 3’ L1Hs primer, 4.5 μM of locus-specific primer, and 200nM of 3’ L1Hs probe was used. Droplet generation was performed as per the manufacturer’s instructions. Cycling conditions were 95°C for 10 min, followed by 40 cycles of 94°C for 30 seconds and 64°C for one minute, and then a final 10-min incubation at 98°C. Droplet reading was performed on a QX200 ddPCR droplet reader (Bio-Rad; Hercules, CA, USA), and analysis was done using QuantaSoft Analysis software (Bio-Rad; Hercules, CA, USA).

Genomic DNA from the following cell lines was used: GM01630, GM01631 and GM01632 (Coriell Institute; Camden, NJ, USA), Flp-In-293 (denoted HEK293 in figures and figure legends, the parental line of these cells; Invitrogen now Life Technologies; Grand Island, NY, USA), HeLa (American Type Culture Collection; Manassas, VA, USA, item number: CCL-2), LoVo (American Type Culture Collection; Manassas, VA, USA, item number: CCL-229), HCT116D (HCT116 derivative with a Flp-In site integrated kindly provided by J. Issa [35]; denoted HCT116 in figures and figure legends).

### Droplet digital PCR mixing experiments

For mixing experiments, cell line genomic DNA positive for a particular L1 insertion was mixed via tenfold dilutions with cell line genomic DNA negative for that particular L1 insertion. Following mixing, dilutions were added at 150 ng to 200 ng per ddPCR reaction as described above.

## Additional files

Additional file 1: Figure S1. Detection of Chromosome 4 esv3475 L1Hs by the 5’ junction ddPCR assay. The L1Hs 5’ junction ddPCR assay uses a L1-specific primer, L1-specific 5’-FAM labeled Taqman™ probe, and a locus-specific primer near the Chromosome 4 esv3475 5’-insertion junction, as shown in Figure 1A. The FAM fluorescent signal (Ch 1) for each droplet is plotted on the y-axis for each ddPCR experiment, which are separated by a dotted yellow line and indicated above each experiment with the input DNA. Each droplet is cumulatively counted as an “Event Number” for the ddPCR experiments analyzed in tandem, and plotted along the x-axis. The positive droplet fluorescence threshold for each fluorophore used is indicated by the magenta line, which determines whether a droplet is considered positive or negative for FAM fluorescence. Thus, the blue dots represent individual droplets that contain at least one copy of the L1 locus tested. We tested 200 ng of *BamHI*-digested genomic DNA from LoVo cells, which contain the polymorphic L1 element, and tenfold dilutions of this same sample as a mixture with *BamHI*-digested genomic DNA from HEK293 cells, which do not have this polymorphic L1 insertion, thus keeping the input genomic DNA constant for each ddPCR. Percentages given reflect the amount of input DNA with 100% corresponding to 200 ng of DNA. This assay robustly detects the 5’-insertion junction of the polymorphic full-length esv3475 L1Hs element when present in the genomic DNA from a cell line positive for that polymorphism (LoVo 100%), but not in a cell line negative for that polymorphism (HEK293 100%). L1-positive droplets are observed at dilutions as low as 0.1% of the DNA for this locus. Additional file 2: Figure S2. Concentration plot of Chromosome 4 esv3475 L1Hs by the 5’ junction ddPCR assay. The input DNA concentrations in copies/μl (Ch1 Conc) for the ddPCR experiments described in Additional file 1: Figure S1 were calculated by the QuantaSoft Analysis Software. Additional file 3: Figure S3. Detection of Chromosome 4 esv3475 L1Hs by the 3’ junction ddPCR assay. The L1Hs 3’ junction ddPCR assay used a L1-specific primer, L1-specific 5’-FAM labeled Taqman™ probe, and a locus-specific primer near the Chromosome 4 esv3475 3’-insertion junction, as shown in Figure 1B. The FAM fluorescent signal (Ch 1) for each droplet is plotted on the y-axis for each ddPCR experiment, which are separated by a dotted yellow line and indicated above each experiment with the input DNA. Each droplet is cumulatively counted as an “Event Number” for the ddPCR experiments analyzed in tandem, and plotted along the x-axis. The positive droplet fluorescence threshold is indicated by the magenta line, which determines whether a droplet is considered positive or negative for FAM fluorescence. Thus, the blue dots represent individual droplets that contain at least one copy of the L1 locus tested. We tested 200 ng of *BamHI*-digested genomic DNA from LoVo cells, which contain the polymorphic L1 element, and ten-fold dilutions of this same sample as a mixture with *BamHI*-digested genomic DNA from HEK293 cells, which do not have this polymorphic L1 insertion. Percentages given reflect the amount of input DNA with 100% corresponding to 200 ng of DNA. The positive droplet fluorescence threshold is indicated by the magenta line. This assay robustly detects the 3’-insertion junction of the polymorphic full-length esv3475 L1Hs element when present in the genomic DNA from a cell line positive for that polymorphism (LoVo 100%), but not in a cell line negative for that polymorphism (HEK293 100%). L1-positive droplets are observed at dilutions as low as 0.1% of the DNA for this locus. Additional file 4: Figure S4. Concentration plot of Chromosome 4 esv3475 L1Hs by the 3’ junction ddPCR assay. The input DNA concentrations in copies/ μl (Ch1 Conc) for the ddPCR experiments described in Additional file 3: Figure S3 were calculated by the QuantaSoft Analysis Software. Additional file 5: Figure S5. Detection of Chromosome 4 esv4912 L1Hs by the 5’ junction ddPCR assay. Experiments were performed as in Additional file 1: Figure S1 to determine the limit of detection for the 5’ junction of the polymorphic full-length esv4912 L1Hs element on Chromosome 4 in a cell line positive for that polymorphism (HCT116 100%), in indicated experiments as an input mixture with genomic DNA from a cell line negative for that polymorphism (HeLa). Additional file 6: Figure S6. Concentration plot of Chromosome 4 esv4912 L1Hs by the 5’ junction ddPCR assay. The input DNA concentrations in copies/μl (Ch1 Conc) for the ddPCR experiments described in Additional file 5: Figure S5 were calculated by the QuantaSoft Analysis Software. Additional file 7: Figure S7. Detection of Chromosome 4 esv4912 L1Hs by the 3’ junction ddPCR assay. Experiments were performed as in Additional file 3: Figure S3 to determine the limit of detection for the 3’ junction of the polymorphic full-length esv4912 L1Hs element on Chromosome 4 in a cell line positive for that polymorphism (HCT116 100%), in indicated experiments as an input mixture with genomic DNA from a cell line negative for that polymorphism (HeLa). Additional file 8: Figure S8. Concentration plot of Chromosome 4 esv4912 L1Hs by the 3’ junction ddPCR assay. The input DNA concentrations in copies/μl (Ch1 Conc) for the ddPCR experiments described in Additional file 7: Figure S7 were calculated by the QuantaSoft Analysis Software.

## Abbreviations

ddPCR: droplet digital PCR
HTS: high-throughput sequencing
L1: long interspersed element 1
qPCR: quantitative PCR
TPRT: target primed reverse transcription

## References

[1] Lander ES, Linton LM, Birren B, Nusbaum C, Zody MC, Baldwin J, Devon K, Dewar K, Doyle M, FitzHugh W, Funke R, Gage D, Harris K, Heaford A, Howland J, Kann L, Lehoczky J, LeVine R, McEwan P, McKernan K, Meldrim J, Mesirov JP, Miranda C, Morris W, Naylor J, Raymond C, Rosetti M, Santos R, Sheridan A, Sougnez C (2001). Initial sequencing and analysis of the human genome. Nature.

[2] Brouha B, Schustak J, Badge RM, Lutz-Prigge S, Farley AH, Moran JV, Kazazian HH (2003). Hot L1s account for the bulk of retrotransposition in the human population. Proc Natl Acad Sci U S A.

[3] Baillie JK, Barnett MW, Upton KR, Gerhardt DJ, Richmond TA, De Sapio F, Brennan PM, Rizzu P, Smith S, Fell M, Talbot RT, Gustincich S, Freeman TC, Mattick JS, Hume DA, Heutink P, Carninci P, Jeddeloh JA, Faulkner GJ (2011). Somatic retrotransposition alters the genetic landscape of the human brain. Nature.

[4] Ewing AD, Kazazian HH (2010). High-throughput sequencing reveals extensive variation in human-specific L1 content in individual human genomes. Genome Res.

[5] Ewing AD, Kazazian HH (2011). Whole-genome resequencing allows detection of many rare LINE-1 insertion alleles in humans. Genome Res.

[6] Helman E, Lawrence ML, Stewart C, Sougnez C, Getz G, Meyerson M (2014). Somatic retrotransposition in human cancer revealed by whole-genome and exome sequencing. Genome Res.

[7] Iskow RC, McCabe MT, Mills RE, Torene S, Pittard WS, Neuwald AF, Van Meir EG, Vertino PM, Devine SE (2010). Natural mutagenesis of human genomes by endogenous retrotransposons. Cell.

[8] Lee E, Iskow R, Yang L, Gokcumen O, Haseley P, Luquette LJ, Lohr JG, Harris CC, Ding L, Wilson RK, Wheeler DA, Gibbs RA, Kucherlapati R, Lee C, Kharchenko PV, Park PJ (2012). Landscape of somatic retrotransposition in human cancers. Science.

[9] Pitkanen E, Cajuso T, Katainen R, Kaasinen E, Valimaki N, Palin K, Taipale J, Aaltonen LA, Kilpivaara O (2014). Frequent L1 retrotranspositions originating from TTC28 in colorectal cancer. Oncotarget.

[10] Shukla R, Upton KR, Muñoz-Lopez M, Gerhardt DJ, Fisher ME, Nguyen T, Brennan PM, Baillie JK, Collino A, Ghisletti S, Sinha S, Iannelli F, Radaelli E, Dos Santos A, Rapoud D, Guettier C, Samuel D, Natoli G, Carninci P, Ciccarelli FD, Garcia-Perez JL, Faivre J, Faulkner GJ (2013). Endogenous retrotransposition activates oncogenic pathways in hepatocellular carcinoma. Cell.

[11] Solyom S, Ewing AD, Rahrmann EP, Doucet T, Nelson HH, Burns MB, Harris RS, Sigmon DF, Casella A, Erlanger B, Wheelan S, Upton KR, Shukla R, Faulkner GJ, Largaespada DA, Kazazian HH (2012). Extensive somatic L1 retrotransposition in colorectal tumors. Genome Res.

[12] Harris CR, Normart R, Yang Q, Stevenson E, Haffty BG, Ganesan S, Cordon-Cardo C, Levine AJ, Tang LH (2010). Association of nuclear localization of a long interspersed nuclear element-1 protein in breast tumors with poor prognostic outcomes. Genes Cancer.

[13] Marusyk A, Polyak K (2010). Tumor heterogeneity: causes and consequences. Biochim Biophys Acta.

[14] Hindson BJ, Ness KD, Masquelier DA, Belgrader P, Heredia NJ, Makarewicz AJ, Bright IJ, Lucero MY, Hiddessen AL, Legler TC, Kitano TK, Hodel MR, Petersen JF, Wyatt PW, Steenblock ER, Shah PH, Bousse LJ, Troup CB, Mellen JC, Wittmann DK, Erndt NG, Cauley TH, Koehler RT, So AP, Dube S, Rose KA, Montesclaros L, Wang S, Stumbo DP, Hodges SP (2011). High-throughput droplet digital PCR system for absolute quantitation of DNA copy number. Anal Chem.

[15] Sykes PJ, Neoh SH, Brisco MJ, Hughes E, Condon J, Morley AA (1992). Quantitation of targets for PCR by use of limiting dilution. Biotechniques.

[16] Pekin D, Skhiri Y, Baret JC, Le Corre D, Mazutis L, Salem CB, Millot F, El Harrak A, Hutchison JB, Larson JW, Link DR, Laurent-Puig P, Griffiths AD, Taly V (2011). Quantitative and sensitive detection of rare mutations using droplet-based microfluidics. Lab Chip.

[17] Holland PM, Abramson RD, Watson R, Gelfand DH (1991). Detection of specific polymerase chain reaction product by utilizing the 5′––3′ exonuclease activity of Thermus aquaticus DNA polymerase. Proc Natl Acad Sci U S A.

[18] Livak KJ (1999). Allelic discrimination using fluorogenic probes and the 5′ nuclease assay. Genet Anal.

[19] Whale AS, Huggett JF, Cowen S, Speirs V, Shaw J, Ellison S, Foy CA, Scott DJ (2012). Comparison of microfluidic digital PCR and conventional quantitative PCR for measuring copy number variation. Nucleic Acids Res.

[20] Roberts CH, Jiang W, Jayaraman J, Trowsdale J, Holland MJ, Traherne JA (2014). Killer-cell immunoglobulin-like receptor gene linkage and copy number variation analysis by droplet digital PCR. Genome Med.

[21] Kidd JM, Graves T, Newman TL, Fulton R, Hayden HS, Malig M, Kallicki J, Kaul R, Wilson RK, Eichler EE (2010). A human genome structural variation sequencing resource reveals insights into mutational mechanisms. Cell.

[22] Boissinot S, Chevret P, Furano AV (2000). L1 (LINE-1) retrotransposon evolution and amplification in recent human history. Mol Biol Evol.

[23] Ovchinnikov I, Rubin A, Swergold GD (2002). Tracing the LINEs of human evolution. Proc Natl Acad Sci U S A.

[24] MacDonald JR, Ziman R, Yuen RK, Feuk L, Scherer SW (2014). The database of genomic variants: a curated collection of structural variation in the human genome. Nucleic Acids Res.

[25] Wang J, Wang W, Li R, Li Y, Tian G, Goodman L, Fan W, Zhang J, Li J, Zhang J, Guo Y, Feng B, Li H, Lu Y, Fang X, Liang H, Du Z, Li D, Zhao Y, Hu Y, Yang Z, Zheng H, Hellmann I, Inouye M, Pool J, Yi X, Zhao J, Duan J, Zhou Y, Qin J (2008). The diploid genome sequence of an Asian individual. Nature.

[26] Stjernqvist S, Ryden T, Greenman CD (2011). Model-integrated estimation of normal tissue contamination for cancer SNP allelic copy number data. Cancer Inform.

[27] Abyzov A, Mariani J, Palejev D, Zhang Y, Haney MS, Tomasini L, Ferrandino AF, Rosenberg Belmaker LA, Szekely A, Wilson M, Kocabas A, Calixto NE, Grigorenko EL, Huttner A, Chawarska K, Weissman S, Urban AE, Gerstein M, Vaccarino FM (2012). Somatic copy number mosaicism in human skin revealed by induced pluripotent stem cells. Nature.

[28] Mullighan CG, Phillips LA, Su X, Ma J, Miller CB, Shurtleff SA, Downing JR (2008). Genomic analysis of the clonal origins of relapsed acute lymphoblastic leukemia. Science.

[29] Navin N, Krasnitz A, Rodgers L, Cook K, Meth J, Kendall J, Riggs M, Eberling Y, Troge J, Grubor V, Levy D, Lundin P, Månér S, Zetterberg A, Hicks J, Wigler M (2010). Inferring tumor progression from genomic heterogeneity. Genome Res.

[30] Parisi F, Ariyan S, Narayan D, Bacchiocchi A, Hoyt K, Cheng E, Xu F, Li P, Halaban R, Kluger Y (2011). Detecting copy number status and uncovering subclonal markers in heterogeneous tumor biopsies. BMC Genomics.

[31] Sabeti PC, Varilly P, Fry B, Lohmueller J, Hostetter E, Cotsapas C, Xie X, Byrne EH, McCarroll SA, Gaudet R, Schaffner SF, Lander ES, Frazer KA, Ballinger DG, Cox DR, Hinds DA, Stuve LL, Gibbs RA, Belmont JW, Boudreau A, Hardenbol P, Leal SM, Pasternak S, Wheeler DA, Willis TD, Yu F, Yang H, Zeng C, Gao Y, International HapMap Consortium (2007). Genome-wide detection and characterization of positive selection in human populations. Nature.

[32] International HapMap 3 Consortium (2010). Integrating common and rare genetic variation in diverse human populations. Nature.

[33] Neve B, Froguel P, Corset L, Vaillant E, Vatin V, Boutin P (2002). Rapid SNP allele frequency determination in genomic DNA pools by pyrosequencing. Biotechniques.

[34] Li-Sucholeiki XC, Tomita-Mitchell A, Arnold K, Glassner BJ, Thompson T, Murthy JV, Berk L, Lange C, Leong-Morgenthaler PM, MacDougall D, Munro J, Cannon D, Mistry T, Miller A, Deka C, Karger B, Gillespie KM, Ekstrøm PO, Todd JA, Thilly WG (2005). Detection and frequency estimation of rare variants in pools of genomic DNA from large populations using mutational spectrometry. Mutat Res.

[35] Zhang Y, Shu J, Si J, Shen L, Estecio MR, Issa JP (2012). Repetitive elements and enforced transcriptional repression co-operate to enhance DNA methylation spreading into a promoter CpG-island. Nucleic Acids Res.

