# Supplementary figures and images for: A droplet digital PCR detection method for rare L1 insertions in tumors
Source: Mob DNA. 2014 Dec 31;5:30. doi: 10.1186/s13100-014-0030-4 (PMC4297411; doi:10.1186/s13100-014-0030-4)

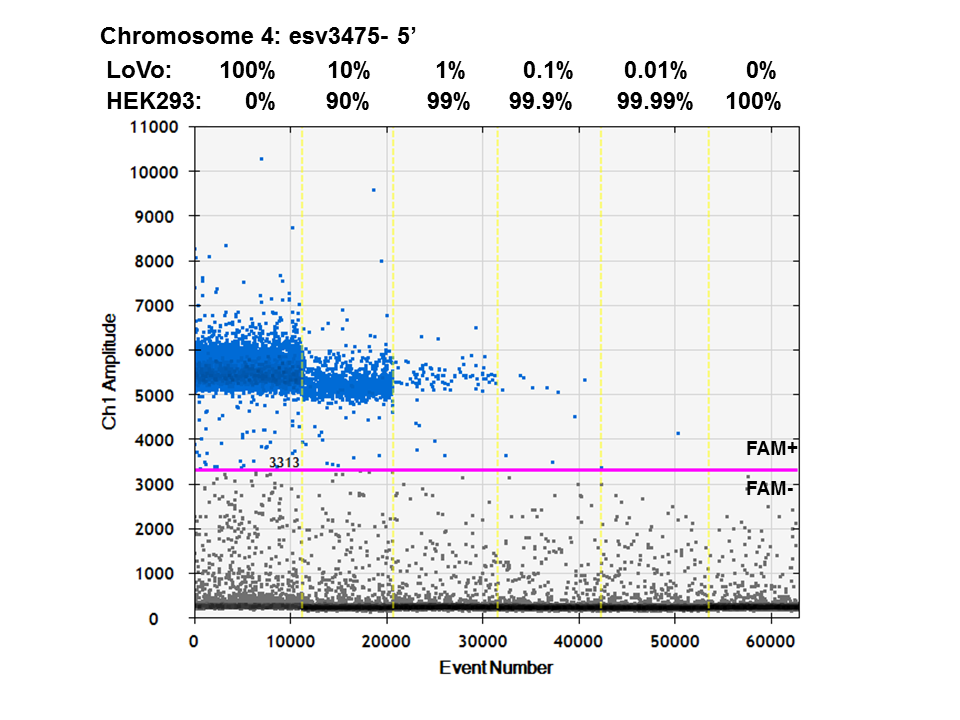

Supplement: Additional file 1: Figure S1. — Detection of Chromosome 4 esv3475 L1Hs by the 5’ junction ddPCR assay. The L1Hs 5’ junction ddPCR assay uses a L1-specific primer, L1-specific 5’-FAM labeled Taqman™ probe, and a locus-specific primer near the Chromosome 4 esv3475 5’-insertion junction, as shown in Figure 1A. The FAM fluorescent signal (Ch 1) for each droplet is plotted on the y-axis for each ddPCR experiment, which are separated by a dotted yellow line and indicated above each experiment with the input DNA. Each droplet is cumulatively counted as an “Event Number” for the ddPCR experiments analyzed in tandem, and plotted along the x-axis. The positive droplet fluorescence threshold for each fluorophore used is indicated by the magenta line, which determines whether a droplet is considered positive or negative for FAM fluorescence. Thus, the blue dots represent individual droplets that contain at least one copy of the L1 locus tested. We tested 200 ng of BamHI-digested genomic DNA from LoVo cells, which contain the polymorphic L1 element, and tenfold dilutions of this same sample as a mixture with BamHI-digested genomic DNA from HEK293 cells, which do not have this polymorphic L1 insertion, thus keeping the input genomic DNA constant for each ddPCR. Percentages given reflect the amount of input DNA with 100% corresponding to 200 ng of DNA. This assay robustly detects the 5’-insertion junction of the polymorphic full-length esv3475 L1Hs element when present in the genomic DNA from a cell line positive for that polymorphism (LoVo 100%), but not in a cell line negative for that polymorphism (HEK293 100%). L1-positive droplets are observed at dilutions as low as 0.1% of the DNA for this locus. [file 13100_2014_30_MOESM1_ESM.tiff]

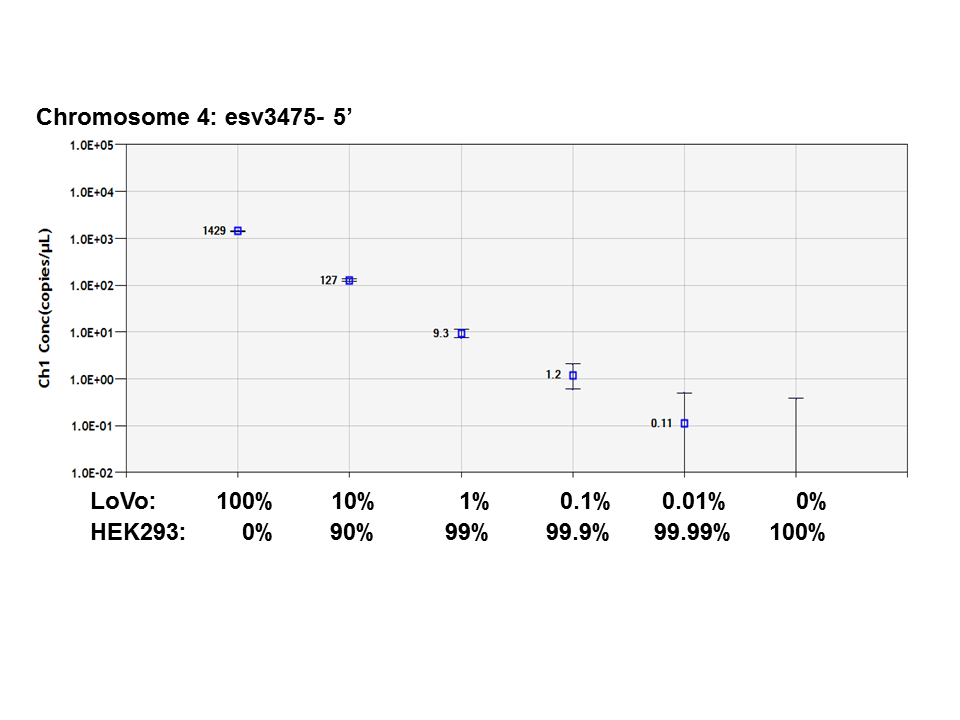

Supplement: Additional file 2: Figure S2. — Concentration plot of Chromosome 4 esv3475 L1Hs by the 5’ junction ddPCR assay. The input DNA concentrations in copies/μl (Ch1 Conc) for the ddPCR experiments described in Additional file 1: Figure S1 were calculated by the QuantaSoft Analysis Software. [file 13100_2014_30_MOESM2_ESM.tiff]

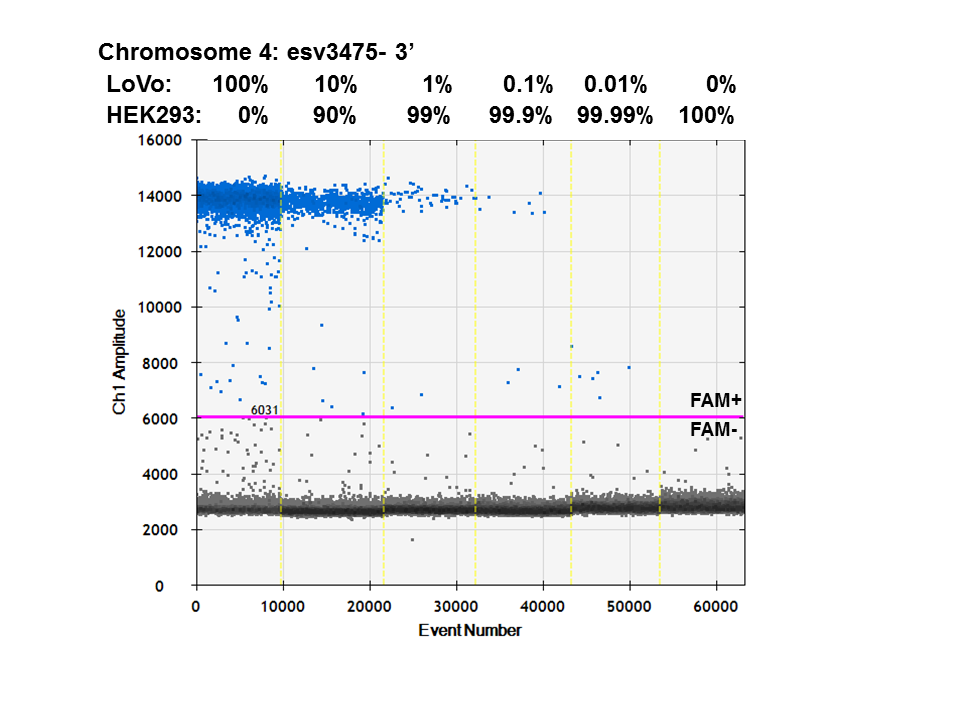

Supplement: Additional file 3: Figure S3. — Detection of Chromosome 4 esv3475 L1Hs by the 3’ junction ddPCR assay. The L1Hs 3’ junction ddPCR assay used a L1-specific primer, L1-specific 5’-FAM labeled Taqman™ probe, and a locus-specific primer near the Chromosome 4 esv3475 3’-insertion junction, as shown in Figure 1B. The FAM fluorescent signal (Ch 1) for each droplet is plotted on the y-axis for each ddPCR experiment, which are separated by a dotted yellow line and indicated above each experiment with the input DNA. Each droplet is cumulatively counted as an “Event Number” for the ddPCR experiments analyzed in tandem, and plotted along the x-axis. The positive droplet fluorescence threshold is indicated by the magenta line, which determines whether a droplet is considered positive or negative for FAM fluorescence. Thus, the blue dots represent individual droplets that contain at least one copy of the L1 locus tested. We tested 200 ng of BamHI-digested genomic DNA from LoVo cells, which contain the polymorphic L1 element, and ten-fold dilutions of this same sample as a mixture with BamHI-digested genomic DNA from HEK293 cells, which do not have this polymorphic L1 insertion. Percentages given reflect the amount of input DNA with 100% corresponding to 200 ng of DNA. The positive droplet fluorescence threshold is indicated by the magenta line. This assay robustly detects the 3’-insertion junction of the polymorphic full-length esv3475 L1Hs element when present in the genomic DNA from a cell line positive for that polymorphism (LoVo 100%), but not in a cell line negative for that polymorphism (HEK293 100%). L1-positive droplets are observed at dilutions as low as 0.1% of the DNA for this locus. [file 13100_2014_30_MOESM3_ESM.tiff]

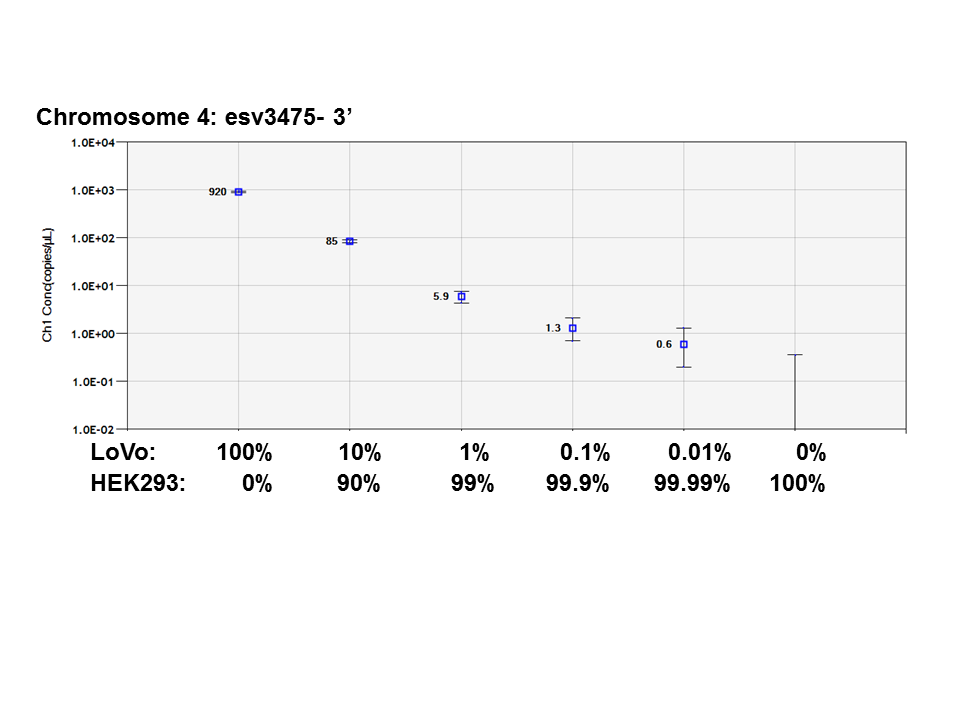

Supplement: Additional file 4: Figure S4. — Concentration plot of Chromosome 4 esv3475 L1Hs by the 3’ junction ddPCR assay. The input DNA concentrations in copies/ μl (Ch1 Conc) for the ddPCR experiments described in Additional file 3: Figure S3 were calculated by the QuantaSoft Analysis Software. [file 13100_2014_30_MOESM4_ESM.tiff]

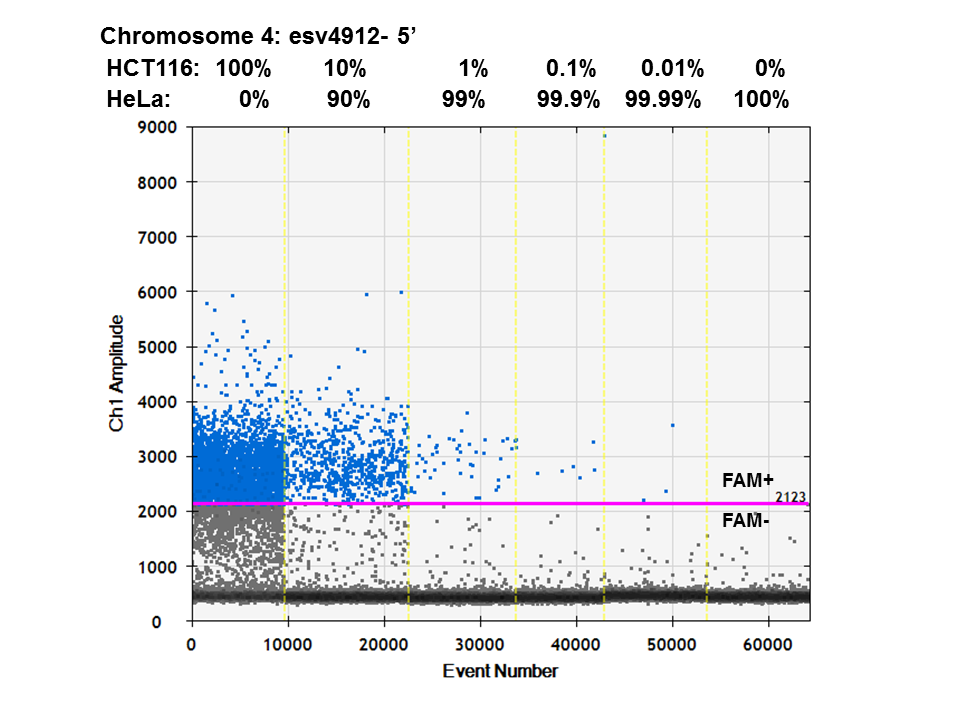

Supplement: Additional file 5: Figure S5. — Detection of Chromosome 4 esv4912 L1Hs by the 5’ junction ddPCR assay. Experiments were performed as in Additional file 1: Figure S1 to determine the limit of detection for the 5’ junction of the polymorphic full-length esv4912 L1Hs element on Chromosome 4 in a cell line positive for that polymorphism (HCT116 100%), in indicated experiments as an input mixture with genomic DNA from a cell line negative for that polymorphism (HeLa). [file 13100_2014_30_MOESM5_ESM.tiff]

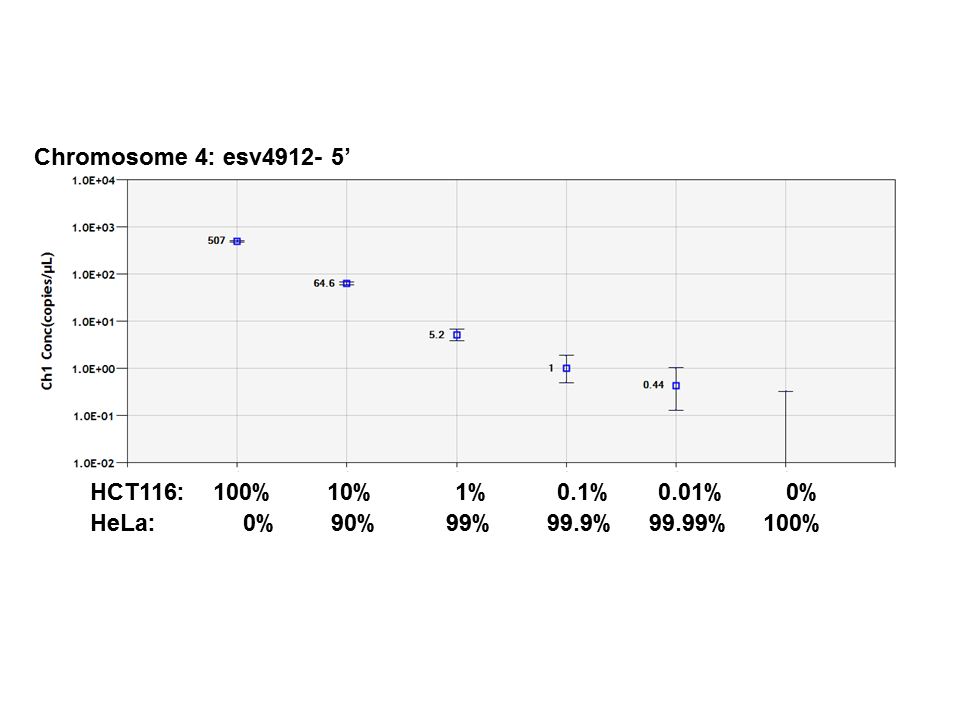

Supplement: Additional file 6: Figure S6. — Concentration plot of Chromosome 4 esv4912 L1Hs by the 5’ junction ddPCR assay. The input DNA concentrations in copies/μl (Ch1 Conc) for the ddPCR experiments described in Additional file 5: Figure S5 were calculated by the QuantaSoft Analysis Software. [file 13100_2014_30_MOESM6_ESM.tiff]

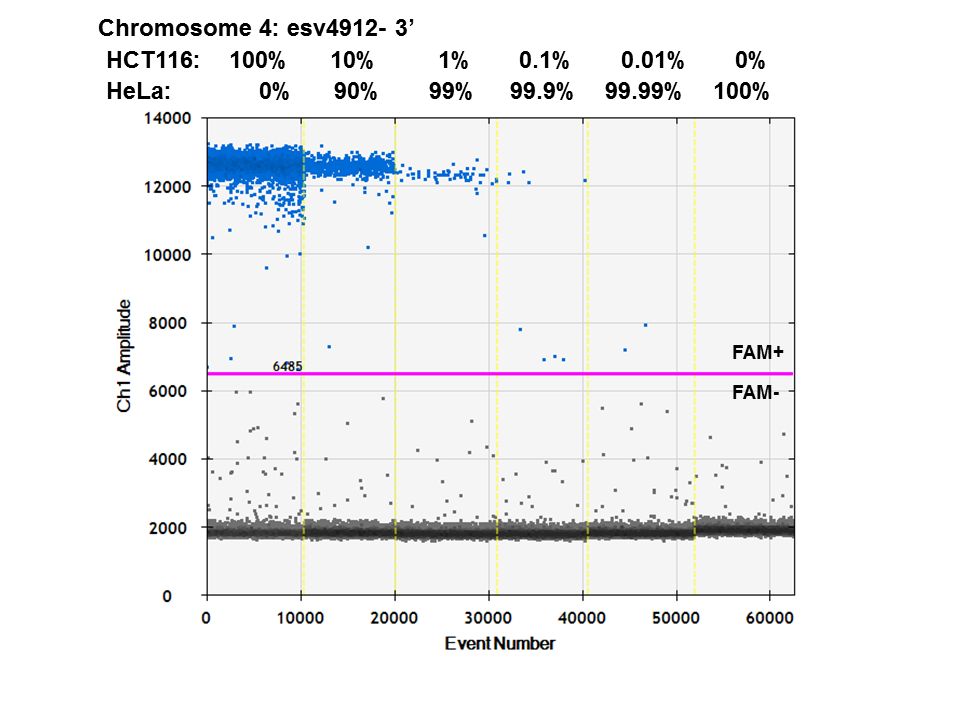

Supplement: Additional file 7: Figure S7. — Detection of Chromosome 4 esv4912 L1Hs by the 3’ junction ddPCR assay. Experiments were performed as in Additional file 3: Figure S3 to determine the limit of detection for the 3’ junction of the polymorphic full-length esv4912 L1Hs element on Chromosome 4 in a cell line positive for that polymorphism (HCT116 100%), in indicated experiments as an input mixture with genomic DNA from a cell line negative for that polymorphism (HeLa). [file 13100_2014_30_MOESM7_ESM.tiff]

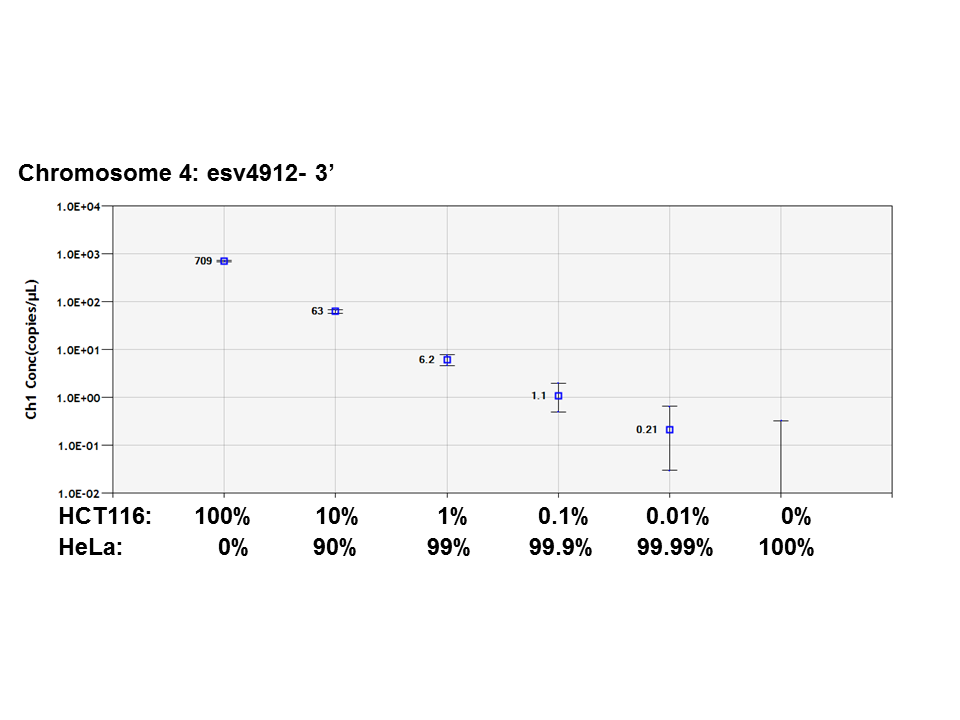

Supplement: Additional file 8: Figure S8. — Concentration plot of Chromosome 4 esv4912 L1Hs by the 3’ junction ddPCR assay. The input DNA concentrations in copies/μl (Ch1 Conc) for the ddPCR experiments described in Additional file 7: Figure S7 were calculated by the QuantaSoft Analysis Software. [file 13100_2014_30_MOESM8_ESM.tiff]
